# Single-session music therapy reduces anxiety and depression among hospitalized adults in an endocrinology ward

**DOI:** 10.1016/j.clinsp.2026.100922

**Published:** 2026-04-10

**Authors:** Veronique Lima, Izabel Cristina Rios, Marlene Inacio, Vinicius Nahime Brito, Berenice B. Mendonca

**Affiliations:** a Department of Endocrinology, Faculdade de Medicina, Hospital das Clínicas da Universidade de São Paulo, São Paulo, SP, Brazil; b Technical and Scientific Center for Humanization (NTH), Faculdade de Medicina, Universidade de São Paulo, Hospital das Clínicas, São Paulo, SP, Brazil

**Keywords:** Music therapy, Single session, Anxiety, Depression, Hospitalized adult, Mixed diagnostics

## Abstract

**Introduction:**

Hospitalization can generate stress and anxiety and lead to depression. Music therapy has been used in hospital environments to promote patient well-being. However, scarce research has examined heterogeneous groups using the single-session format. This controlled clinical trial aimed to compare the effects of three methods of single-session music therapy on reducing anxiety and depression in hospitalized adult patients with diverse endocrine disorders.

**Method:**

Participants (*n* = 222) were non-randomly distributed into three experimental music therapy groups (compositional, songwriting, and receptive) and a control group. The instruments used were the Hospital Anxiety and Depression Scale (HADS; pre-and post-test).

**Results:**

The experimental groups presented a statistically significant decrease in the HADS scores (*p* < 0.05) compared with the control group. Participants in the experimental groups reported an enhancement in well-being.

**Discussion:**

Single-session music therapy interventions, mainly the receptive method, had a positive impact on reducing anxiety and depression in a heterogeneous group of hospitalized adult patients with endocrinopathies.

## Highlights

• Music therapy has a positive impact on adult patients with different endocrinopathies.
• Music therapy decreases anxiety and depression in hospitalized patients.
• A single session of music therapy enables patients to reconnect with their sense of self.
• •Music therapy improves hospital adjustment for patients with endocrinopathies.

## Introduction

Hospitalization is an event that generates anxiety, stress, and depression.^1^^,^^2^ Depression may be aggravated owing to patients being distant from their family, home, and daily routine.^3^ Social isolation, reduced mobility, and disease severity also lead to increased anxiety and depression during hospitalization.^4^ Consequently, anxiety and depression may hinder individuals’ ability to cope with diseases, negatively impact treatment, worsen patients’ physical condition, and increase the number of bedridden days.^5^ However, the short duration of physician consultations and limited privacy in many hospital environments hinder private conversations between patients and doctors. As a result, these emotional states may often pass by undetected.

These mood disorders may evoke emotional reactions (e.g., anger, noncompliance, irritability) that jeopardize treatment during hospitalization. Therefore, coping strategies to support patients in dealing with their emotions during this period are fundamental for their well-being.^6^

In this context, music therapy (MT) can be an effective strategy, but it is important to emphasize the difference between MT and music medicine. In MT, interventions are conducted by a trained music therapist who uses specific qualities of music and creates a therapeutic relationship with clients, while music medicine is basically the experience of listening to music offered by medical or healthcare professionals.^7^^,^^8^ Many studies have demonstrated the effectiveness of music medicine in reducing anxiety in hospitalized patients^9^^,^^10^ and improving moods.^11^

MT has been used in hospital environments to promote well-being and reduce stress^12^^,^^13^ anxiety^14^ and pain.^15^ The use of MT in healthcare is very promising;^16^ most MT research in the hospital environment is conducted with groups of patients with homogenous diagnoses undergoing MT for 5–52 sessions.^17^, ^18^, ^19^ However, so far, little research has been conducted in heterogeneous groups concerning diagnostics using single-session MT.

The term single-session has been derived from psychotherapy research; single-session psychotherapy is widely used in major catastrophes, emergencies, and humanitarian situations, mainly owing to the enormous number of people that are affected, thereby leading most people to be able to participate in only one therapy session.^20^

Endocrinology is a specialty that takes care of disorders of endocrine glands. Most of the patients were hospitalized in the short term to perform endocrinology tests or correct metabolic disorders; all participants from this study were inpatients. Those patients have to deal with the stress caused by the hospitalization itself. As they also have chronic diseases, it is common for them to feel anxious about their health. Chronic diseases are a risk factor for the advent of anxiety and depression.^21^^,^^22^ MT could help to prevent these problems, reducing anxiety and alleviating depressive symptoms, even with a small number of sessions.^22^

There is no information in the literature regarding the comparison of three different MT approaches with endocrinology patients in a single session. The aim is to compare three MT approaches to identify the best way to perform a single session with these patients, helping them with non-medication therapy. The authors’ questions are: can single-session MT reduce anxiety and depression in hospitalized endocrinology patients? What type of single-session MT gives better results for these cohorts?

## Methods

### Ethical considerations

This study was approved by the Ethics Committee for Analysis of Research Projects (CAPPesq) on December 13, 2017, under protocol number 2.433.642. Approval for human subjects’ research was obtained, and all participants signed an informed consent form.

### Participants

The number of patients in the infirmary varied per week, so it was impossible to know how many patients would be hospitalized in advance. Therefore, throughout the weeks, the participants were non-randomly distributed consecutively in the three experimental MT groups (compositional, songwriting, and receptive MT), and the control group.

**Fig. 1:**
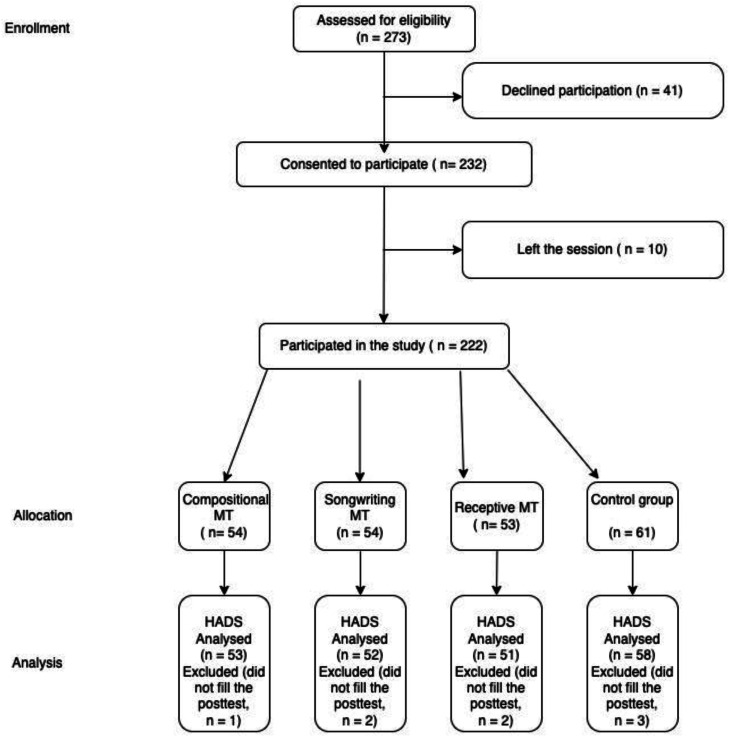
CONSORT Sample recruitment and group allocation flowchart.

The authors invited 273 patients (i.e., who were 18-years or older and hospitalized in the endocrinology ward of the University of São Paulo, School of Medicine Hospital das Clinicas) to participate; 41 opted out, 2 abandoned the session, and 8 were taken out of the session for medical procedures. Therefore, 222 patients participated and had their data analyzed (Fig. 1).

Non-participation or session abandonment did not jeopardize patients’ treatment in the hospital in any way, and all participants were reassured of this. Participants received no payment for participation, and hospital patients were allowed to participate in the sessions without being study participants. In order to obtain homogeneity, the experimental and control groups comprised patients who did not have any cognitive alteration, severe pain, or surgery on the day of the session, and were willing to attend. The mean time of hospitalization was 5.9 to 8.2 days.

### Design

This trial was conducted according to the guidelines outlined in the CONSORT statement.^23^ This is a four-arm parallel design study. Patients were non-randomly distributed into four groups as follows: Three experimental (i.e., compositional, songwriting, and receptive MT) and one control group (i.e., hospitalized patients who did not undergo any MT session). The interventions of MT were based on Daniel Stern’s theory, in which intersubjectivity is defined as a basic need in the social context and its absence generates anxiety, causing the appearance of coping and defense mechanisms.^24^ Therefore, the focus of the MT group sessions was to facilitate intersubjectivity contact in order to reduce anxiety.

One of the researchers, a qualified music therapist, developed the study protocols prior to study onset based on a pilot group. These protocols were based on different MT approaches and created especially for hospitalized endocrinology patients.

Every week, the authors invited hospitalized patients to participate in this study in the following order: the first week for songwriting MT, the second week for compositional MT, the third week for receptive MT, and the fourth week for the control group. This process was repeated until the authors reached the desired number of participants. The control group was a masked group; namely, patients were invited to participate as a control group in a survey about stress during hospitalization. The control group did not receive an intervention. These patients simply completed the HADS and followed their usual hospital routine. The real name of the study was not declared to avoid bias. The first investigator instructed patients to fill out and hand-deliver the HADS to the healthcare team by the end of the day, just as patients in the MT groups did.

### Procedure

The researcher entered patients’ rooms and invited them to participate in one of the groups, depending on the week, one group per week. If they agreed, written informed consent was obtained. All patients filled out the pre-test HADS. The post-test HADS was filled within seven days after the pre-test. Because the scale asks how the patient felt during the last week, the participant was instructed to think about the previous few days and not just in the present moment. Then, the idea was that they would have a few days between the first and the second application. The control group was analyzed under the same conditions. Each MT session for the experimental groups lasted for 50–60 minutes.

The MT sessions focused on the process, rather than the final product (i.e., the composition, lyrics, and drawing). This process was explained to patients, helping them feel more secure about participating, knowing that there would be no aesthetic judgment of their work.

In order to ensure fidelity to the research, session protocols were developed, which were read by the music therapist before each session. There were also regular meetings with the interventionist and other members of the research team, aiming to reduce differences and avoid deviations from the study protocols.

### Data collection

All data were collected from December 2017 to October 2019. The HADS was validated in Brazil in 1995^25^ and the authors used it owing to its efficiency in measuring anxiety and depression and its brevity. Additionally, it was easy to comprehend and answer. It comprises 14 multiple-choice questions divided into two subscales: anxiety (HAD-anxiety; 7 items) and depression (HAD-depression; 7 items). Item score ranges from 0 to 3 points, and total scores for each subscale range from 0 to 21 points. Zigmond and Snaith^26^ classified the scores as follows: 0–8 = without anxiety or depression (depending on the subscale); ≥ 9 = with anxiety or depression. Patients were asked about their feelings during the last week, within the scope of the requirements of single-session MT.

### Sessions’ description

The interventions were group-based; they took place in the meeting room (little ambient sound, high privacy) located inside the endocrinology ward every Monday afternoon, from 5:00–6:00 PM., comprising activities that encouraged active participation. The authors used the following materials in the sessions: recorded audio material, songs suggested by the researcher, speakers, musical instruments (i.e., wood shaker, bell stick, triangle, agogo, guiro, xylophone, drum, and mini keyboard), sheets of paper, pencils, and crayons. The sound volume was controlled by the music therapist.

All sessions began with the group listening to a “chorinho” (popular Brazilian instrumental music) song called “Papagaio embriagado” by Mascarenhas.^27^

Patients in all three experimental groups reported the sensations and/or images evoked by this music. This first stage was meant to engage participants in a condition that allowed them to reflect upon their own sensations. Afterwards, the three types of MT (i.e., songwriting, compositional, and receptive) were developed differently, as described below.

The songwriting session comprised the creation of lyrics based on the melody of the music “Asa Branca”, a well-known national song composed by Luiz Gonzaga. At the start of the session, participants were asked to talk about their sensations and feelings regarding their hospitalization experience and to create a written list of the positive and negative aspects. After creating this list, patients were invited to write new lyrics for the song “Asa Branca” by including the negative aspects of hospitalization in the first two verses of the song and the positive aspects in the third and fourth verses. When the lyrics were ready, everyone sang the new version created by the group. On the same day, the music therapist delivered a copy of the lyrics to all participants. At the end of the research, the authors sent a list with all the negative aspects of the hospital environment to the hospital ombudsman.

The compositional session involved the creation of music based on a drawing. First, patients were divided into two subgroups; the therapist remarked that each subgroup should freely choose a song without letting the other subgroup know about the music of choice. Subsequently, each subgroup made a free drawing based on any aspect of the chosen song. After finalizing the drawings, the subgroups exchanged them. The therapist invited each subgroup to compose a musical piece based on the other subgroup’s drawing, with the help of any of the instruments placed on the table, their own voices, or other sounds, such as using their hands or feet to create rhythm. In the end, each group presented its composition, played it, explained how they interpreted the drawing, and how they transformed it into music for the other subgroup. The main purpose of this session was to give patients back their autonomy, allowing them to be the authors of their actions by being involved in the composition process. In the receptive session, patients were invited to listen to the song “Evening quiet” by Lanfranco Perini.^28^ While listening to this song, participants were requested to close their eyes and, with verbal guidance by the researcher, let themselves be transported by their imagination to an enjoyable place. To facilitate this imaginary journey, the music therapist turned the lights off. When the music ended, participants were invited to make an individual drawing based on this experience. After finishing the drawing, each participant was invited to tell the group how they felt about the event.

**Fig. 2:**
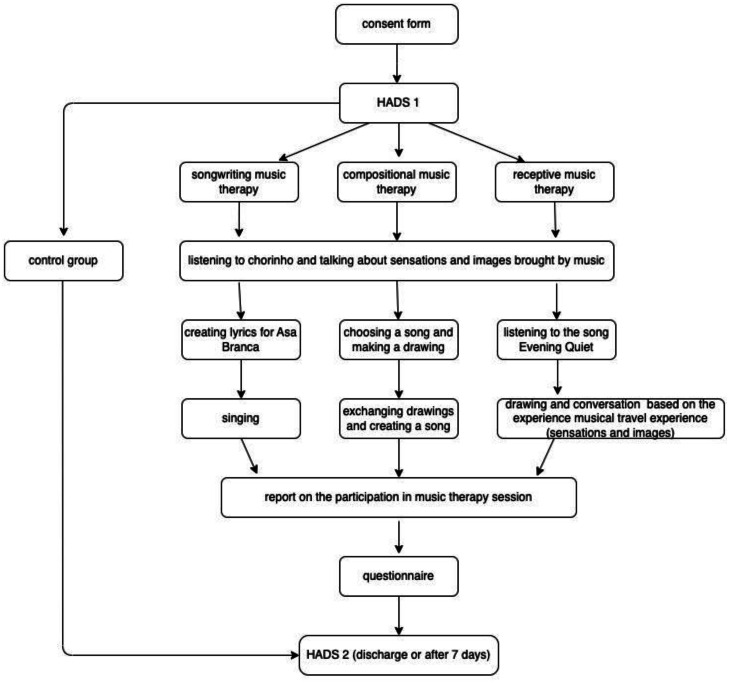
Research process flowchart.

At the end of each MT session, participants talked about their sensations and feelings regarding the session (Fig. 2).

### Statistical analysis

Statistical analyses were performed using IBM SPSS Statistics for Macintosh, Version 27.0. The recommended sampling size was 212 patients; this was calculated based on the average number of patients admitted annually in the Endocrinology ward (i.e., 462 patients), a sampling error of 5 %, a confidence interval of 95 %, and a heterogeneous population^29^ Normally distributed variables were presented as means and standard deviations, while non-normally distributed variables were presented as medians and ranges. The Kolmogorov-Smirnov test was used to test the null hypothesis that a set of data comes from a normal distribution. Intra-groups pre- and post-intervention scores comparisons were made by Student’s paired *t*-test or Wilcoxon test according to normal or non-normal distribution, respectively. The intergroup comparisons were run using one-way analysis of variance (ANOVA). Categorical variables were presented as simple percentages and compared using the chi-square test.

In an additional analysis, the dependent variables (anxiety or depression) were considered as binary variables (yes/no) and the differences in proportions in patients pre-and post- MT intervention were assessed by the two-tailed McNemar’s Chi-Square test. Statistical significance was set at *p* < 0.05. To assess the magnitude of the intervention's effect, Cohen's *d* was calculated for the entire cohort, comparing the pre- and post-intervention scores. Cohen's *d-*value of 0.2 was interpreted as a small effect, 0.5 as a moderate effect, and 0.8 as a large effect.

## Results

Participants comprised 155 women and 67 men; their mean age was 46.6 ± 16.4 (18–88.4). There were no statistically significant differences between the MT and control groups with respect to the distribution of age and gender. The average number of participants consisted of three people per session/week; in total, there were 72-weeks of sessions, 18 for each group. Patients had several endocrine diseases (some with more than one diagnosis). The most prevalent condition was type I and type II diabetes mellitus in all groups (Table 1).

**Table 1:** Distribution of endocrine diseases in Music-Therapy (MT) groups and control.

Endocrine Diseases	CMT	SMT	RMT	C
Type I and type II diabetes mellitus	12	12	16	12
Parathyroid diseases (hyperparathyroidism, hypoparathyroidism)	8	6	7	6
Thyroid diseases (thyroid nodules, hypothyroidism, hyperthyroidism, nontoxic and plunging goiter)	8	9	4	7
Multiple endocrine neoplasia type I and II	4	6	5	9
ACTH-dependent or independent Cushing's syndrome	7	6	5	5
Non-functioning pituitary tumors or producers of GH, prolactin or ACTH	1	5	4	9
Hyperaldosteronism	2	2	1	7
Syndromic conditions	3	0	4	0
Tumors or lesions of the adrenal gland, congenital adrenal hyperplasia	8	4	2	4
Hypoglycemia	1	1	1	3
Pheochromocytomas and paraganglioma	0	1	6	0
Pancreatic tumors	0	2	2	2
Differences in sexual development 46,XY	1	2	0	1
Dyslipidemia	1	1	1	0
Morbid obesity	1	0	2	1
Transsexuality	0	1	0	1
Diabetes insipidus	0	0	0	1
Pancreatitis	0	0	0	1

CMT, Compositional MT; SMT, Songwriting MT; RMT, Receptive MT; C, Control.

### HADS

The mean interval of the HADS application (1‒7 days) in the MT groups did not differ from that in the control group. The first HADS test was applied before the MT session, and the second HADS was applied after a median time of 4 days (one day in 13.9 %; 2 days in 16.6 %; 3 days in 15.7 %; 4 days in 44.1 %; 5 days in 3.6 %; 6 days in 0.4 %, and 7 days in the remaining 1.8 %).

In the compositional group, there was no statistically significant difference between the pre- and post-intervention mean scores for the HADS-Anxiety (HADS-A) and HADS-Depression (HADS-D). However, there was a significant difference between the pre- and post-intervention median of the HADS-Total score (HADS-T) (*p* = 0.022; Table 2).

**Table 2:** Scores of the HADS at pre- and post-intervention in MT groups and controls.

Parameter	Group	Pre-intervention	Post-intervention	p		
		n	score	n	score	
HADS-A	Compositional MT	54	8.18 ± 3.98^a^	53	7.39 ± 4.0	0.062
	Songwriting MT	54	8.59 ± 4.9	52	7.6 ± 4.59	0.073
	Receptive MT	53	8 (1‒16)^b^	51	6 (0‒18)	0.006^c^
	Control	61	6 (0‒16)	58	5 (0‒17)	0.5
HADS-D	Compositional MT	54	6 (0‒15)	53	5 (0‒13)	0.17
	Songwriting MT	54	6 (1‒16)	52	5 (0‒16)	0.015^c^
	Receptive MT	53	7 (0‒14)	51	6 (0‒15)	0.028^c^
	Control	61	5 (0‒14)	58	4 (0‒16)	0.068
HADS-T	Compositional MT	54	14 (1‒29)	53	13 (0‒31)	0.022^c^
	Songwriting MT	54	15.18 ± 7.37	52	13.23 ± 7.52	0.004^c^
	Receptive MT	53	16 (2‒25)	51	13 (1‒32)	0.001^c^
	Control	61	11 (1‒30)	58	10 (0‒33)	0.17

HADS, Hospital Anxiety and Depression Scale; MT, Music Therapy.

a Mean ± SD.

b Median (range).

c p < 0.05. The combined Cohen's *d* for anxiety (0.19) and depression (0.16) was 0.18, indicating a small effect of the intervention on reducing anxiety and depression among participants.

In the songwriting group, there was no statistically significant difference between the pre- and post-intervention mean scores for HADS-A; however, there was a significant decrease between the pre- and post-intervention mean scores for the HADS-D (*p* = 0.015), and between the pre- and post-intervention mean scores for the HADS-T (*p* = 0.004) (Table 2).

In the receptive group, there was a significant decrease between pre- and post- intervention values in all parameters (i.e., HADS-A, *p* = 0.006; HADS-D, *p* = 0.028 and for the HADS-T, *p* = 0.001; Table 2).

Importantly, in the control group, there was no significant difference between the pre- and post-intervention for the HADS-A and HADS-D, and between the pre- and post-intervention median of the HADS-T score (Table 2).

Interestingly, the authors observed a higher initial mean score for both subscales in the experimental MT groups compared with those in the control group, without a statistically significant difference (*p* > 0.05). All MT groups and controls showed a decrease in mean scores post-intervention.

Additionally, the authors analyzed the percentage of patients who presented a decrease in HADS-A score pre-and post-intervention, and it was greater in the MT than in the control group. In the compositional group, 29 participants (54.7 %) showed a reduced post-intervention compared with pre-intervention; 26 participants (50 %) in the songwriting group; 31 participants (60.78 %) in the receptive group; and 27 participants (46.5 %) in the control group. Some patients presented an increase in the score for the HADS-A post-intervention compared with pre-intervention. This occurred in 26.4 % of the participants in the compositional, 28.8 % participants in the songwriting, 27.4 % participants in the receptive, and 39.6 % of the participants in the control group.

The percentage of patients who presented a decrease in HADS-A score pre-and post- intervention was also greater in the MT than in the control group. This happened in 60.3 % of the compositional MT, 55.7 % of the songwriting MT, 58.8 % of the receptive MT group, and 41.3 % of the control group. Some patients presented an increase in the score of HADS-D post- intervention compared with pre-intervention. This occurred in 28.3 % of the participants in the compositional, 28.8 % participants in the songwriting, 27.4 % participants in the receptive, and 34.4 % participants in the control group.

Finally, considering anxiety or depression (HADS cut-off score ≥ 9) as binary variables (yes/no), the proportion of patients who presented anxiety or depression at baseline and changed after the intervention was significantly higher only in the receptive MT (*p* = 0.02 and 0.01 for anxiety and depression, respectively) (Table 3).

**Table 3:** Comparisons between number of patients with anxiety and depression before and after intervention in MT groups and controls.

The comparison of mean delta HADS scores (i.e., the difference between pre- and post-intervention values) across endocrine diagnostic subgroups (diabetes, thyroid, parathyroid, adrenal, pituitary disorders, or miscellaneous conditions) revealed no significant differences.

This suggests that the specific endocrine diagnosis did not influence the intervention's outcomes. Overall, the intervention led to a small but measurable reduction in anxiety and depression symptoms, with a combined Cohen’s *d* of 0.18, indicating a modest yet positive effect across both domains.

### Patient comments

After MT sessions, patients were encouraged to write anonymous comments in the absence of the music therapist. All patients declared that they felt good about participating in the MT group session and that it improved their well-being. Of the 222 participants, 98 included comments (i.e., 33 from the compositional MT group, 43 from the songwriting MT group, and 22 from the receptive MT group) and 63 did not; the remaining 61 belonged to the control group, who did not participate in the MT sessions. There were some recurring statements in patients’ comments, such as the perception that the session had a playful aspect; this was expressed by words such as “unwinding”, “feeling like a child”, “relaxing”, and “recreation”. As positive aspects of the session, they remarked that it provided them with a possibility to “forget the suffering”, “relieve the anguish”, “have a moment of happiness”, “relax”, “feel at ease”, “feel at peace”, and “feel relief”. According to the comments, patients saw MT as something that positively influenced their feelings and minds when experiencing significant physical and mental vulnerability. The sessions also helped these patients relive good memories and provided some moments of relief from tension, during which they could even forget that they were in a hospital.

Some affirmed that MT changed the way they were feeling, how they were experiencing hospitalization and their perception of themselves. It was also observed that MT helped them to remind their identities as human beings, since some of the participants had declared that before intervention, they could only see themselves as patients.

### Discussion

This research evaluated the effect of a single session comparing different methods of MT (i.e., compositional, songwriting, and receptive MT) in a heterogeneous group of adult patients with various endocrine disorders hospitalized in an endocrinology ward.

The authors observed that the MT sessions had a positive effect on diminishing participants’ anxiety and depression levels, evaluated by the HADS score. There was a statistically significant decrease in anxiety and depression in the receptive MT group. These results could be explained by the fact that listening to music increases dopamine production, generating well-being.^30^ From a physical and cognitive standpoint, receptive music therapy demands less of patients, which may explain the present results. Compared to the other groups, the receptive music therapy group showed the best results, corroborating the idea that adult hospitalized patients generally prefer receptive music therapy interventions.^31^^,^^32^ However, the songwriting music therapy group's results were very similar, suggesting that well-balanced active interventions that engage patients without overwhelming them may be beneficial in a hospital setting.

In this study, the second application of the HADS occurred from one to seven days after the MT session. This prolonged effect has also been found in other studies using receptive MT^33^^,^^34^ and single sessions.^35^ Another example is the McKinney study using Guided Imagery and Music (GIM) with 28 healthy participants, with sessions every 2-weeks. The GIM sessions significantly reduced depressed moods and the levels of serum cortisol, and the results were maintained for at least 7-weeks after the end of the sessions.^34^

The authors also observed MT session benefited from the participants' comments. The patients reported that the session provided them with a moment where they felt seen, heard, welcome, and secure. Some participants also stated that the MT session helped them reflect on the moment they were experiencing, while also helping them elucidate and express their emotions toward hospitalization.

One of the characteristics of the MT sessions that could have led participants to feel welcome may relate to the songs utilized; all MT sessions started with a well-known Brazilian music style (chorinho) that has a strong association in participants’ daily lives. According to Postacchinni et al.^36^ using this kind of musical material in the initial stages of an integration project is important. Similarly, an initial intervention with familiar songs helps create a therapeutic alliance in patients’ psychological elaboration process.^37^

Single sessions can help people reconnect with their sense of self^35^ which can be helpful for patients during hospitalization, and this factor has probably influenced the reduction in levels of anxiety and depression. Additionally, MT sessions allowed patients to make choices, so they could feel they had some control over the situation, thus reducing the symptoms of stress.^38^

It further promoted communication among participants of the MT group, thereby increasing patient–patient and patient–healthcare team integration and helping them better adapt to the hospital environment. Supporting these results, a study demonstrated a positive impact of MT on patients’ mood and patient–healthcare team interactions.^39^

Intersubjectivity is a basic need in the social context, and its absence generates anxiety, causing the appearance of coping and defense mechanisms. Thus, reducing anxiety in patients may be related to the fact that MT sessions provide this intersubjective contact.^24^

The existence of a control group and the fact that the sessions were conducted by the same interventionist (greater homogeneity between groups) contributed to better internal validity. The sample size, with consistent power calculation, is also a factor that contributed to a greater external validity of the research.

The present study had some limitations. There is a potential bias owing to the dual role played by the principal investigator, who was both the researcher and music therapist. Future studies should use independent therapists. The non-randomization and consecutive allocation of participants introduce bias; however, it was not possible to access in advance information about the number and the names of patients who would be admitted. Access to this information was only granted on the day of the interventions. Despite being included on the list of potential participants on the day of the study, many patients were unable to participate due to their subsequent transfer to other departments for various diagnostic procedures. The scheduling of these tests is not dictated by a predetermined schedule; rather, it is contingent upon the workflow of the hospital. Consequently, the duration required to reach the stipulated sample size for the study could not be ascertained (i.e., months or years). In subsequent studies, a randomized sequence for the allocation of groups prior to each month could be implemented.

This is the first study showing that a single-session MT in a large cohort of patients with endocrinopathies results in positive achievement, opening this opportunity for other hospitalized cohorts. Another important aspect is that there are few music therapy studies on endocrine disorders. The inclusion of patients with diverse chronic endocrinopathies marks a significant departure from studies focusing on single conditions, thereby broadening the scope and applicability of the research to a wider patient population.^40^ Furthermore, the exploration of different music therapy approaches allows for a comparative assessment, aiming to identify the most effective therapeutic strategy for this specific group of patients, potentially paving the way for personalized treatment protocols.^41^ Due to the benefits of the MT groups, it could be implemented in more public hospitals as part of the patient care service, with the music therapist being part of the health team. There is a need to create a safe and welcoming space for adults that provides a sense of belonging and a feeling of enormous importance during hospitalization.

In conclusion, single-session MT interventions, mainly the receptive MT, had a positive impact in reducing anxiety and depression in a heterogeneous group of hospitalized adult patients with endocrinopathies.

## Data statement

All data are available within the text.

## Data availability

Data not available due to ethical/legal/commercial restrictions.

## Funding

This research received no specific grant from any funding agency in the public, commercial, or not-for-profit sectors.

## Declaration of competing interest

The authors declare no conflicts of interest.
